# Cultural Influences on African Migrant Pregnant and Postnatal Women’s Dietary Behaviours and Nutrition Support Needs in the UK

**DOI:** 10.3390/nu15194135

**Published:** 2023-09-25

**Authors:** Lem Ngongalah, Tim Rapley, Judith Rankin, Nicola Heslehurst

**Affiliations:** 1 Population Health Sciences Institute, Newcastle University, Newcastle upon Tyne NE2 4AX, UK; judith.rankin@newcastle.ac.uk (J.R.); nicola.heslehurst@newcastle.ac.uk (N.H.); 2 Department of Social Work, Education and Community Wellbeing, Northumbria University, Newcastle upon Tyne NE7 7XA, UK; tim.rapley@northumbria.ac.uk

**Keywords:** overweight, obesity, nutrition, pregnancy, preconception, African, migrant, culture, women

## Abstract

Black women in the UK face significantly higher risks of overweight and obesity and adverse pregnancy outcomes compared to women from other ethnic groups. Maternal nutrition plays a pivotal role in influencing the health outcomes of women and their children, especially during preconception and pregnancy. Cultural and environmental factors significantly influence the dietary experiences of African women after migration. This study explored the unique nutrition-related challenges faced by African migrant pregnant and postnatal women in the UK, and their nutrition support needs. Interviews were conducted with 23 African migrant women living in the UK, who were either pregnant or had a pregnancy within the past 3 years. These were analysed thematically, resulting in five overarching themes: food rituals and beliefs, pregnancy cravings, limited access to culturally appropriate food, limited access to culturally appropriate and evidence-based nutritional guidance, and the focus on healthy weight. The study identified challenges that African migrant women face in balancing their cultural heritage with the UK food environment and dietary recommendations, including potential implications on their health and pregnancy outcomes. It emphasised the importance of addressing these challenges through culturally sensitive approaches and tailored interventions, to enable informed decision making and enhance health outcomes for these women.

## 1. Introduction

Overweight and obesity among women of reproductive age pose a significant public health concern in the UK, due to their prevalence and potential impacts on maternal and infant health [1,2]. Maternal overweight and obesity are associated with various pregnancy complications, including an increased risk of gestational diabetes mellitus (GDM), hypertension, preterm birth, stillbirths and caesarean deliveries [3,4,5,6]. These conditions not only impact the wellbeing of the mother but also contribute to potential complications for the newborn, such as macrosomia, congenital anomalies, and a higher likelihood of developing obesity and associated health issues later in life [3,7,8,9,10]. An analysis of weight status among women from diverse ethnic backgrounds in the UK showed that two-thirds (66.6%) of Black women had overweight or obesity in early pregnancy, compared to 48.6% of White women, 22.8% of women with Chinese ethnicity, 51.8% of Asian women and 51.2% of women with mixed ethnicities [11]. Black women also face a five-fold higher likelihood of dying from complications during pregnancy and childbirth compared to women from other ethnic backgrounds in the UK [12]. The causes of these disparities are multi-factorial, including socioeconomic inequalities, inadequate access to quality healthcare, pre-existing health conditions and systemic bias [13]. These disparities necessitate targeted efforts to understand and address the multifaceted factors contributing to the observed inequalities in this population. 

Maternal nutrition plays a pivotal role in influencing the health outcomes of women and their children, especially during preconception and pregnancy [14]. Adequate nutrition is essential to sustain the mother’s energy levels during pregnancy and to support the growing demands of the developing foetus [14]. The preconception period also presents an opportunity to support women to adopt healthier behaviours in preparation for a successful pregnancy and positive health outcomes both for themselves and their children [11,14]. This includes the postnatal period, which is an extension of the preconception period due to its potential impact on future pregnancies [15]. Inadequate nutrition during preconception or in pregnancy can lead to increased susceptibility to various pregnancy-related complications and poor birth outcomes, including difficulties during labour and delivery, maternal mortality, low birth weight, preterm birth and neural tube defects [14,16]. These findings underscore the crucial role of preconception and periconception nutrition in shaping maternal and fetal health outcomes, as nutrition in these early stages provides the vital nutrients necessary for optimal growth and development. As part of the Commission on Ending Childhood Obesity, the World Health Organisation recognised preconception and pregnancy care as 1 of 6 key areas for action, calling for clear guidance and support for the promotion of good nutrition and dietary counselling in antenatal care [17]. This body of evidence emphasises the importance of providing appropriate nutrition support and guidance to women of child-bearing age, particularly those identified to be at higher risk of overweight, obesity, and adverse pregnancy outcomes.

While Black women in the UK may share some common experiences, it is important to acknowledge that within this population, there exists a wide range of cultural backgrounds, traditions, and lived experiences. African migrant women often bring with them unique dietary practices, culinary traditions, and cultural beliefs related to food and nutrition from their countries of origin, which can play a crucial role in shaping their dietary behaviours and nutritional needs. In addition, the ability for African migrant women to access healthy foods and adopt healthy dietary behaviours in the UK is influenced by a combination of pre- and post-migration factors [18], as well as social and environmental factors including socioeconomic status, language barriers, access to healthcare and social support, and acculturation processes [19,20,21]. This study aimed to explore the unique nutrition-related challenges faced by African migrant pregnant and postnatal women in the UK, and their nutrition support needs. The findings from this study could provide valuable insights to inform targeted interventions that can offer practical nutrition support for these women and help reduce the risks related to inadequate nutrition during this life course period including obesity, pregnancy-related complications, and maternal mortality. Understanding these cultural and environmental influences is also crucial for improving diet quality and ensuring adequate nutrition for subsequent pregnancies among African migrant women.

## 2. Materials and Methods

### 2.1. Study Participants and Recruitment

This qualitative study was conducted in England, UK, and targeted African migrant women from Nigeria, Ghana, and Cameroon, who were aged 18–45 years and had been living in England for a minimum of 6 months. Eligible participants were either pregnant at the time of the study or had been pregnant in England within the last 3 years. Participant recruitment employed a comprehensive approach, combining purposive, convenience, and snowball sampling methods. Purposive sampling involved targeting women through community groups and organisations that cater to African populations in England, such as churches, non-governmental organisations, and support groups. Information about the study and researcher contact details were disseminated through flyers to these organisations, and interested participants initiated contact. Three social media platforms (Facebook, WhatsApp, and Twitter) were also used to reach out to African groups online. Study details and contact information were circulated on these platforms to attract potential participants. Convenience sampling was employed by leveraging personal networks to recruit eligible women for the study and snowballing involved participants referring other potentially eligible individuals within their networks.

### 2.2. Data Collection

Data collection was carried out through semi-structured interviews, some in person and others virtually. In-person interviews were arranged at locations convenient to participants, such as their homes or quiet public spaces, to ensure a comfortable and private environment that upheld ethical considerations and maintained strict confidentiality. Participants provided written consent before each in-person interview, while those interviewed virtually provided consent through email and verbal confirmation. Interviews were guided by a topic guide (Appendix A), which consisted of open-ended questions to encourage discussion. The topic guide was pilot-tested and continuously refined throughout the interview process to further explore issues discussed in earlier interviews. Interviews were conducted between November 2018 and May 2019. All interviews were recorded using an encrypted digital recorder, and the data were securely stored on a secure network at Newcastle University, following the University’s Information Security Guidelines [22] and the General Data Protection Regulation [23].

### 2.3. Data Analysis

Interviews were transcribed and anonymised by assigning identification numbers (e.g., P1) to participants to maintain confidentiality. Anonymised interviews were analysed using thematic analysis, following the guidelines provided by Braun and Clarke [24]. This process involved repeated reading of the transcripts to gain familiarity with the data, assigning initial codes to developing ideas, and organising similar codes into themes. The coding process was carried out by one author (LN) and resulting codes and themes were discussed with the wider team to ensure consensus and enhance the rigor of the analysis. Data were managed and coded using NVivo (version-12) [25].

### 2.4. Presentation of Study Findings

The resulting themes and subthemes are presented in Section 3, offering a thorough exploration of the cultural factors influencing the dietary behaviours and nutritional support needs of African migrant women. Results from the study are integrated with the broader literature to contextualise and provide a more comprehensive understanding of the findings. Illustrative quotes are provided to exemplify the participants’ perspectives and experiences. To maintain participant anonymity, longer person-specific quotes are identified by participant identification numbers, while commonly held views or terms expressed by multiple participants are not linked to specific identifiers (embedded in text in italics). Quotation marks are used in text to denote specific terms used by participants. Long pauses are indicated using ellipses, while any necessary clarifications or contextual information is enclosed within square brackets.

## 3. Results

This study involved 23 women whose ages ranged from 23 to 41 and had lived in England for an average of 6.8 years. Most women were either married or cohabiting (*n* = 19), had two or more children (*n* = 15), and had achieved a university-level education or higher (*n* = 16). Seventeen participants were employed at the time of the study. Five participants were pregnant during the interview, with three being pregnant for the first time. Nine women had a pregnancy in the last year, while five had children aged 1–2 years, and four women had children aged 2–3 years. The study resulted in five overarching themes: food rituals and beliefs, pregnancy cravings, limited access to culturally appropriate food, limited access to culturally appropriate and evidence-based nutritional guidance, and the focus on healthy weight.

### 3.1. Food Rituals and Beliefs

(i) Food taboos and restrictions

Various African cultures associate certain foods with beneficial effects for women’s preconception and during pregnancy, while others are considered taboo due to beliefs about their potential harmful effects on the mother and the unborn child [26,27,28,29,30,31]. Foods believed to promote fertility, vitality, and overall wellbeing are often encouraged for pregnant women, because they are believed to “*enable you carry your pregnancy successfully and give birth to a strong child*”. Meanwhile, some foods such as snails, eggs, beans, and some types of fish and meat are avoided because “*they can cause complications when you want to give birth*”. One participant offered some insights into her culture’s practices:

“In our culture, they advise women not to eat snails when they are pregnant because there’s this belief that when you eat snails—you know snails are very slow when they move…so the idea is that eating snails can make the baby to be lazy, and they would be—their development would be slow”.

(P7)

In addition to specific foods, certain fruits like pineapples were restricted in some cultures during pregnancy “*because it might lead to premature labour or other complications*”. While some of these beliefs had causal explanations grounded in either everyday reasoning or more supernatural theories, others did not follow a clear pattern and were difficult to explain. Reflecting on this, a participant shared:

“I remember discussing this with my mother. I realised that there are lots of traditional things that we cannot really explain, and sometimes they may not even make sense to you, but we believe them because they’ve been there through different generations, and all we know is that they’re there for a good reason—to protect us and our children”.

(P13)

The complexity of the cultural context, interwoven with personal experiences and historical narratives, often made it difficult to provide straightforward explanations for these dietary practices and restrictions. Despite the lack of clear rationale, the women continued to practice these traditions, highlighting their deep-rooted significance even when their origins or implications may not be fully understood.

(ii) Food preferences related to gender and physical attributes

Participants shared distinct cultural beliefs related to specific foods and their impact on the physical or gender attributes of their unborn babies. A participant from Nigeria described an example relating to yam and okra:

“I know that many women follow this belief, that when you eat more yam, then you’re more likely to have a healthy and strong baby boy, and if you eat more okro, then you can have a baby girl. My grandmother used to emphasise—because she had 5 boys and 3 girls, so she’ll say it’s because she used to eat those things when she wanted boys or girls. When I was pregnant, I was eating yam, even though maybe not to the extent that they wanted, and I had a baby boy, but I can’t say for sure if yam had anything to do with it. But I know that they tell many women about it and ask them to eat a lot of it”.

(P2)

Yam is a starchy root crop rich in carbohydrates. It is a significant dietary staple in many parts of Africa, providing essential nutrients such as carbohydrates, fibre, potassium, and vitamin C [32]. Meanwhile, okra is a green vegetable rich in dietary fibre, retinol (vitamin A), vitamin C, and minerals like calcium and iron [33]. Other examples include bananas which were believed to “*make your baby to have smooth skin*” while papaya was believed to “*make the baby’s colour [complexion] lighter*”. These beliefs were often based on family anecdotes and personal experiences.

(iii) Eating for two

The misconception that pregnant women need to eat for two is common and can have significant implications for women and their children [34]. Participants identified that this belief is common in African communities and can influence pregnant women to consume excessive amounts of food, believing it to be necessary for a healthy pregnancy.

“I grew up hearing that during pregnancy, you should eat for two to have a healthy baby. So I would eat much more than usual, they would insist…you have to eat for the both of you, you know you’re pregnant, and so on. Then, well you can see I have a big body naturally, so my weight has always been up. When I was pregnant, I also had ermm…gestational diabetes, and then I started learning about the things that can cause it, and that’s how I started thinking maybe it’s not really needed to eat as much, but it’s about eating the right type of food and the right amount for the baby”.

(P13)

While specific nutrient requirements are crucial for maintaining a healthy pregnancy, substantial increases in energy requirements only occur during the third trimester, and the increase is relatively modest [35]. The notion of doubling food intake is therefore not accurate and can lead to overeating and excessive gestational weight gain (GWG), which might result in complications such as GDM and hypertension. Moreover, the “*eating for two*” belief could emphasise quantity over the quality of diets during pregnancy, potentially leading to an inadequate intake of essential nutrients. This perspective was echoed by a participant who, following this belief, encountered issues with iron deficiency:

“In Nigeria, we believe that during pregnancy, the baby takes all the nutrients from the mother, so we need to eat a lot to make sure that the baby and the mother are healthy. So I would mostly eat big portions of heavy food, with lots of starch, many vegetable soups, and even sweet things, juice, drinks…to make sure that the baby gets enough. I then learned after that I’m not getting enough nutrients like iron and ermm…I think it’s calcium”.

(P5)

Starchy foods and vegetable soups, though nutritious, may not provide enough bioavailable iron, which the body more easily absorbs [36,37]. In addition, consuming excessive quantities of beverages, such as juices and drinks, may lead to high levels of free sugar consumption, resulting in increased risks of GDM [38]. 

### 3.2. Pregnancy Cravings

(i) Cravings for energy-dense cultural food

Cravings for cultural food were common experiences among African migrant women, often influenced by “*memories of home*”, a desire to connect with their cultural roots, and a sense of comfort associated with traditional dishes:

“My go-to comfort food is jollof rice. Whenever I feel homesick or stressed, just give me some jollof—problem solved. You cannot go to a Nigerian party and you don’t find jollof, it’s a very special food, and, I don’t know but there’s just this nice feeling when you’re eating your country food”.

(P2)

Jollof rice is a popular dish in many West African countries [39]. Participants described it as “*a taste of home*” attesting to its deep emotional significance and its ability to evoke cherished memories of family gatherings and celebrations. Cravings for various other traditional dishes like fufu, cassava, and plantains were described, often triggered or intensified by factors such as feelings of homesickness, or the stress of adjusting to a new environment.

“We used to eat a lot of cassava and plantains when I was back home. It was very common for us. Then, I came here, and I realised that it’s not so easy to find them. You can’t buy them in the normal supermarkets, so it now became once in a while, you know, when you find it. Right now that I’m talking, I’m even salivating because I have this big craving for plantains—especially the ripe ones. I usually just like to fry or boil it and eat with pepper sauce”.

(P9)

While recognising the cultural and psychological significance of these foods, it is also important to address the nutritional aspects and any potential implications especially if consumed excessively during preconception or in pregnancy. Many of the dishes highlighted tend to fall within the category of energy-dense foods, which may pose risks of obesity, diabetes, and cardiovascular problems [40]. In addition, it is important to note that the cooking techniques used to prepare these dishes often include methods like deep frying, which can further contribute to an elevated intake of unhealthy fats and calories [41,42].

(ii) Cravings for readily available processed foods

Processed foods are more accessible in the UK and can lead to cravings due to convenience and marketing [43]. Cravings for items like fast food or snacks are driven by factors such as their widespread availability, ease of access, and the alignment of their consumption with the swift and demanding pace of the UK lifestyle [43]. While such cravings naturally occur as part of the dietary acculturation process after migration [44], they do give rise to potential concerns regarding the nutritional intake and overall health of women during preconception and pregnancy. For instance, participants identified how shifts in taste preferences and heightened senses during pregnancy can amplify desires for specific foods that they may not have been drawn to previously, and how they satisfy their pregnancy cravings with these items due to convenience:

“I never used to be a fast-food person, I always loved proper food, like a good home-cooked meal. Even if I want to eat outside, it’s maybe once in a while, and it’ll still be some African food in a restaurant. But when you’re staying in a place where everything is rush rush, there’s so many choices around, there’s not much time to be cooking all the time, of course you will gradually go into the fast food habit Then you know, when you’re pregnant it’s almost as if your body develops a mind of its own. You will start craving for things that even your normal self wouldn’t want to eat all the time. I had this crazy craving for [specific fast-food chain] burgers…it was so crazy, like, my husband had to buy it for me everyday after work, and that’s after I’ve probably already had some in the day. Because there’s one [specific fast-food restaurant] on our street, not far from my house. So it was an everyday thing for me. Everybody knew me with that”.

(P10)

The proximity of the fast-food outlet, its convenience as a time-saving option within walking distance, and the fast-paced environment contributed to her gradual shift towards increased fast-food consumption. Participants shared several other examples such as french fries which they found to be “*quick and satisfying*”, and easily became a craving in pregnancy due to the smell and taste which were “*hard to resist*”. Crisps and soft, sweet treats such as gummy candies, donuts and cookies were also cited as common cravings, which women initially thought to be “*too sweet*”, but overtime they transitioned from being occasional indulgences to becoming “*almost irresistible*” because “*they are so tempting*” and “*they are everywhere*”:

“Sometimes you’ll go to an event, and they serve snacks, there’s some crisps so I’ll pick some. Or I’ll go to visit my friend and her children will come to share with me, they’ll say ‘Aunty have some crisps’ and you know, bit by bit, I started getting familiar with the taste. Then pregnancy came…hmm…it felt like that was the only thing I could eat that won’t make me throw up. I had them everywhere, every time, at the train station, on break at work, if I’m lying on the chair at home, I’m driving, that’s how I just kept eating and eating”.

(P2)

### 3.3. Limited Access to Culturally Appropriate Food

African migrant women living in the UK often face challenges in accessing culturally appropriate food that reflects their culinary traditions and preferences, as the new food environment may not always offer the same variety of ingredients or traditional dishes they are accustomed to:

“When you move here it’s basically like stepping into a whole new world. Our food has specific flavours, and we use particular spices to make them, but they are hard to find in the UK. I remember a time when I was trying to make ndole…I will be looking and looking for where to find washed bitterleaf [green leafy vegetable commonly eaten in West Africa] or crayfish…not to talk of something like achu! [traditional Cameroonian dish made from cocoyam] (laughs) It has its own special spices, but you won’t find them here”.

(P23)

Consequently, these women must adopt certain coping strategies to navigate the changes in their dietary behaviours. One such strategy involves actively seeking out African or ethnic grocery stores that import products from their countries of origin thereby granting access to traditional ingredients, spices, and seasonings, enabling them to recreate cherished dishes:

“There are some African, Caribbean and Asian stores that import them and bring here, and sometimes you might be lucky to have some of those in your area, but it’s still a big challenge because first, you may not even find exactly what you’re looking for, and even when you manage to find something you can use, it’s so expensive”.

(P10)

Participants expressed challenges in having to travel long distances to access the ingredients they need due to the scarcity of these stores. Moreover, even when these stores are available, the limited variety of products and the high cost of the imported items further hinder their ability to regularly incorporate these cultural foods into their diets. Participants also disclosed that when certain ingredients are unavailable, they adapt their recipes using locally available substitutes. This flexibility enables them to maintain the essence of their traditional dishes while incorporating new flavours from the UK:

“I’ve embraced the multicultural aspect of living in the UK, so I’ve had to modify some of our recipes by using some ingredients from local supermarkets to try and replicate the flavours of our food. It’s been challenging, but I’ve also discovered that experimenting with different seasonings and spices or herbs can create some new and interesting combinations”.

(P3)

This adaptive approach empowers women to preserve the flavours they hold dear while embracing the diversity of additional tastes. However, these nutritional compromises could pose health risks to women during preconception and pregnancy, as the substitutes often come with elevated levels of unhealthy fats, added sugars, and salt [45]. Examples of these substitutes include instant seasoning mixes as alternatives to traditional seasonings which often contain high levels of salt and artificial flavour enhancers; canned or processed meat such as sausages or bacon which are often high in sodium, saturated fats, and preservatives; dried herb blends which often contain added salt and preservatives; and garlic and onion power which may lack the same nutritional benefits as fresh produce [46]. 

### 3.4. Limited Access to Culturally Appropriate and Evidence-Based Nutritional Guidance

(i) Uncertainty about nutritional needs during pregnancy

Limited access to culturally appropriate nutrition information and inadequate knowledge about appropriate portion sizes and nutrient requirements can be significant challenges faced by African migrant women in the UK, particularly during pregnancy [47]. Due to cultural differences and the absence of familiar resources, these women may struggle to find relevant and reliable nutrition information that aligns with their traditional dietary practices [18,47]. Participants described feeling lost due to encountering diverse dietary advice in the UK and the perceived necessity to modify their established eating habits, which was a challenge due to the lack of clarity concerning the healthiness of alternative food options and their nutritional compositions:

“In my house, we eat a lot of swallows [a category of starchy foods that are prepared in a dough-like consistency and are typically eaten with various types of soups or sauces], like poundo [pounded yam], eba [grated cassava], semo [casava flour], amala [yam flour], with different soups. There could be an issue with some of them, like maybe I need to change how I’m eating them when I’m pregnant, but I can’t do anything about it because I don’t even know what the problem is or whether the food is giving me all the nutrients I need or not. And even if I wanted to substitute with something else here that is more healthy, I still don’t know what to replace with. So, the challenge for me is always that I want to do what’s best for me and also for my baby, but it’s hard when I’m not sure about the nutrients I need or what I can eat to have them”.

(P13)

These findings show the dilemma faced by African migrant women as they grapple with the fusion of their cultural traditions and the practicalities of adapting to a different food environment. The women express their desire to make the best decisions for themselves and their babies, but the lack of clarity on nutrient needs and unfamiliarity with the local food options create uncertainty. They also acknowledge the need for possible adjustments to their habitual diets during pregnancy, but the lack of specific knowledge about nutrient content and healthy substitutes leaves them feeling unsure about the most suitable alternatives. This uncertainty might result in women opting to “*stick to what you know*”, thereby relying on familiar practices which may not necessarily be optimal for women during preconception and in pregnancy.

(ii) Reliance on social networks

Social networks play a crucial role in the distribution of information among African migrant communities in the UK. Women often turn to family members, friends, and community gatherings to seek advice and share experiences relating to diet and nutrition, particularly regarding the complexities in the UK context. For instance, participants detailed how their involvement in online communities or social groups composed of fellow migrants from their countries of origin proved instrumental in discovering alternatives to cultural foods that were unavailable in the UK.

“I’m on two WhatsApp groups where we usually talk about cooking. There’s one that’s about modified recipes, because there are our local ingredients or spices that we cannot find in the UK shops, so we share ideas about what we can use instead. Like if you want to cook eru now, it’s a Cameroonian food, and you don’t have waterleaf, I learned from the women there that you can use spinach. Some women use peanut butter to make groundnut soup, meanwhile normally we would use dry groundnuts. Then I remember one time I was missing garden egg sauce, but I couldn’t find garden eggs in the supermarket where I always go to buy food. So I learnt that you can use aubergine. It tastes very similar”.

(P8)

These virtual platforms served as invaluable resources for sharing tips, recipes, and information about locating specific ingredients. However, participants noted the absence of similar online resources catering specifically to pregnancy-related dietary needs, highlighting a significant gap in addressing their distinct migrant and cultural requirements during this period. A participant recounted how her social networks helped in acquiring pregnancy-specific nutritional advice:

“When I first came to the UK and found out I was pregnant, I felt lost and overwhelmed with all the new information about what to eat and what to avoid. So, I reached out to other Cameroonian women I knew here, and they shared their experiences and what they did during their pregnancies. It was so helpful but also nice to hear from people who understood my situation and shared my experience”.

(P5)

Participants also cited food bloggers as important sources of information on cultural foods during pregnancy. These online influencers were perceived as relatable and empathetic, “*…especially those ones that have been pregnant here and they understand the challenges that we face with our food—you know that they know what they’re talking about” (P21).*

While these social networks offer valuable support and cultural insights, the accuracy and comprehensiveness of the information shared can vary, posing a potential risk of misinformation or creating further uncertainty. 

### 3.5. Focus on Healthy Weight—A Complex Perspective

The focus on healthy weight during pregnancy is a complex subject for African migrant women in the UK, as it involves navigating cultural beliefs, personal priorities, and the influence of their migrant experiences on views about health. These women face challenges in balancing between adhering to their traditional beliefs about weight and health, which are often reinforced by their family and social networks, and integrating new knowledge from their host country’s healthcare system. For instance, gaining weight during pregnancy is typically seen as a positive sign that the baby is growing well. Receiving advice to regulate their weights can therefore be challenging to reconcile with their longstanding beliefs, as it involves negotiations with established family norms:

“Back home, when a woman gains weight during pregnancy, it’s seen as a good thing. People say it means the baby is growing well, and it’s a sign of a healthy pregnancy. So, when I moved to the UK and they started telling me to watch my weight, I was confused. Like, I thought gaining more weight as the baby is growing was a good thing?”

(P9)

Adding a layer of complexity is the process of migrant acculturation and adaption to a new healthcare system. This is further compounded by a spectrum of experiences where economic demands, family responsibilities, and other factors might often take precedence over concerns related to health, such as nutrition or weight status.

“You’re either trying to find work that suits your availability, figuring out how to pay the bills, and for some of us, we have to take care of our families here, and also help our families back home. With all these big things going on, sometimes I don’t really have the energy to be thinking about what I’m eating or what my weight is. It’s not that I don’t care about my health, but that’s not really my priority right now. I’m focused on looking after my family, and work, and handling everything else, that’s what matters most to me right now”.

(P3)

In addition, the challenge of accessing appropriate guidance on suitable nutrition during pregnancy, especially tailored to their African dietary preferences and cultural norms, exacerbates the struggles faced by these women:

“I think it’s important to eat the right things so that me and the baby can be healthy, but it’s very difficult to find advice or recommendations around African food. If I have to be thinking of that then I’ll just be adding another problem to the long list of problems I’m already facing in this country”.

(P11)

The lack of specific guidance on suitable African dietary practices during pregnancy poses an additional obstacle that compounds the existing challenges faced by African migrant women.

## 4. Discussion

This study explored the role of cultural and migrant-related influences on the dietary experiences of African migrant women in the UK, shedding light on how these factors shape their dietary behaviours and nutritional needs during the preconception (including postnatal) period, and in pregnancy. The findings reveal not only the ways in which traditional food practices are adapted and maintained in a new environment, but also the challenges that women face in balancing their cultural heritage with the dietary recommendations provided in the UK. 

Cultural practices such as food taboos and restrictions are a notable concern especially during the crucial phases of preconception and pregnancy, as they can potentially limit the dietary options available to women, contributing to both inadequacies and deficiencies in nutrients required for a healthy pregnancy. For instance, limiting consumption of fish may result in decreased intake of omega-3 fatty acids, which are essential for foetal brain and eye development [48]. Avoiding certain fruits during pregnancy may also mean missing out on essential vitamins and antioxidants, such as vitamin C, which is important for immune support and overall health during pregnancy [49]. The consequences of such dietary restrictions extend beyond nutrient deficiencies. Imbalances in nutrient intake can impact blood sugar regulation, thereby elevating the risk of GDM, with potential health ramifications for both the mother and baby [50]. Imbalances in nutrient intake have also been linked to cravings for energy-dense foods high in sugar and fats, which could lead to overconsumption of less-healthy foods [51]. 

Relying excessively on specific food groups may lead to the neglect of other essential ones, consequently depriving women of diverse nutrients vital for a healthy pregnancy and fetal development. Consuming starchy staples in large quantities during pregnancy, which appeared to be a prevalent behaviour among the women, could potentially contribute to excessive GWG, elevating the risk of complications like gestational hypertension and preeclampsia [52,53]. Excessive okra consumption for perceived gender impacts might hinder iron absorption, potentially increasing the likelihood of iron-deficiency anaemia [54]. This concern is particularly important within this population, as iron-deficiency anaemia is commonplace among women from African countries and significantly affects pregnancy outcomes [55,56]. Prioritising a well-rounded diet encompassing diverse food groups is essential. While promoting adaptations of customary African diets can help bridge this gap, the risks associated with using local food alternatives and substitutes—which are often processed and often contain excessive salts, sugars, and unhealthy fats—are noteworthy. Many processed foods are calorie-dense but nutrient-poor, offering little in terms of essential vitamins, minerals, and other nutrients vital for both maternal health and fetal development [57]. Relying on such foods to satiate cravings can lead to inadequate intake of key nutrients, potentially contributing to adverse pregnancy outcomes. These findings highlight the need for guidance to aid informed dietary decisions for these women. 

Navigating the intersection of healthy nutrition and cultural influences requires an approach marked by nuance and cultural sensitivity. Healthcare professionals can play a pivotal role in facilitating open and respectful communication with African migrant women and providing evidence-based guidance on pregnancy-related nutrition and weight management, while recognising the significance of these cultural influences. Additionally, community-based initiatives, along with tailored resources that acknowledge the diverse perspectives surrounding their health and pregnancy experiences can empower women to adopt healthier dietary behaviours during preconception and in pregnancy. Such culturally tailored interventions have been shown to have significant positive impacts on various obesity-related outcomes in migrant populations including body weight, diet quality, and physical activity [58,59,60,61]. Embracing these multifaceted strategies holds the potential to improve birth outcomes and ensure sustained health benefits for both African migrant women and their children.

Online platforms and social communities host a multitude of perspectives and advice. This diversity serves as both a strength and a challenge—while it fosters a rich exchange in ideas and experiences, it also exposes vulnerabilities to misinformation and conflicting guidance. This issue was evident during the COVID-19 pandemic, when migrant communities turned to social media for information due to accessibility barriers (e.g., language), and misinformation from these sources led to reduced participation in preventive health measures [62]. In this information-rich era, the risk of misconstrued facts, poorly researched recommendations, or personal anecdotes being taken as universal truths is a real issue. While social networks, food bloggers and online communities can offer helpful insights and emotional support, their guidance should ideally be supplemented by advice from healthcare professionals and reputable nutritional sources. Integrating personal experiences, cultural heritage, and medical expertise forms a well-rounded basis for informed choices that prioritise the well-being of women and children.

As migrant and ethnic minority populations in HICs continue to grow, understanding and addressing factors contributing to health disparities becomes increasingly vital. The findings of this study illuminate the critical intersection of cultural influences and dietary practices among African migrant women in the UK, particularly during preconception and pregnancy. To enhance health outcomes in this population, we recommend a multifaceted approach. Firstly, healthcare policies should prioritise cultural sensitivity training for healthcare professionals working with diverse populations. This training will enable them to provide evidence-based guidance on pregnancy-related nutrition and weight management while acknowledging the significance of cultural influences. Secondly, tailored resources and community-based initiatives should be developed, recognising the diverse perspectives and experiences of African migrant women. These initiatives can empower women to adopt healthier dietary behaviours during preconception and pregnancy, aligning with culturally specific interventions that have demonstrated positive effects on various health outcomes. Simultaneously, guidelines should be developed to inform the adaptation of traditional diets, promoting healthy dietary behaviours while respecting cultural identities. However, it is essential to remain vigilant about the potential risks associated with processed food alternatives. It is also crucial to encourage and enable African migrant women to utilise reputable nutritional sources as their primary sources of guidance to ensure informed decision making. 

This study’s strengths include continuous discussions of data interpretations by the research team to ensure the accurate representation of participant views and alignment with the existing literature. Secondly, the shared ethnicity between the principal investigator and participants fostered rapport, while the diverse backgrounds of the wider research team brought varied perspectives. Limitations encompass reliance on participant memory in interviews and potential data loss in theme selection. Nonetheless, the study provides valuable insights into the cultural influences on the dietary experiences and needs of African migrant women.

## Data Availability

The data presented in this study are available on request from the corresponding author. The data are not publicly available due to ethical restrictions.

## Appendix A. Topic Guide

Note: The interview questions encompassed a wide range of topics concerning diet, physical activity, and weight status. This study focuses on the findings relating to dietary behaviours and nutritional support needs

Interview Questions 1. Changes in dietary behaviours after migration. How would you describe the types of food available in the UK and what you eat, compared to when you were back home? Potential areas to discuss further: - Current dietary behaviours—compare England vs. back home. - Changes in dietary patterns after migration—factors associated with these changes. 2. Changes in physical activity (PA) behaviours after migration. How would you describe your PA levels in the UK compared to when you were back home? Potential areas to discuss further: - Current PA behaviours—compare England vs. back home. - Changes in PA behaviours after migration—factors associated with these changes. 3. Dietary behaviours during pregnancy. Did you make any changes to your usual diet during pregnancy? Potential areas to discuss further: - Any changes to their diet when pregnant compared with before pregnancy. - Types of food they ate/avoided. - Factors influencing dietary changes during pregnancy—reasons why they did or did not change their dietary behaviours in pregnancy. - Any difficulties with maintaining a healthy diet. - Compare England vs. back home—if pregnant in African country previously, explore any differences between dietary behaviours in the UK and back home. If not pregnant in African country previously, explore their perceptions on whether their behaviours would have been different if they were back home. 4. PA behaviours during pregnancy. Did you make any changes to your usual daily activities during pregnancy? Potential areas to discuss further: - Any changes to their PA behaviours while pregnant compared with before pregnancy. - Types of activities they did. - Factors influencing dietary changes during pregnancy—reasons why they did or did not change their PA behaviours in pregnancy. - Any difficulties with staying active during pregnancy. - Compare England vs. back home—if pregnant in African country previously, explore any differences between PA behaviours in the UK and back home. If not pregnant in African country previously, explore their perceptions on whether their behaviours would have been different if they were back home. 5. Perceptions on pre-pregnancy weight and weight status in pregnancy. There are several things that could have an effect on a woman or her child during pregnancy, things like smoking, drinking alcohol, taking drugs, being overweight or obese before pregnancy, etc. Are there any of these things that you think are very important to look out for? Potential areas to discuss further: - Which pregnancy risk factors they give priority to and why. - Perceptions about their weights before vs. during pregnancy. - How they think their pre-pregnancy weight could affect their pregnancy. - Whether they think of overweight/obesity as a risk factor in pregnancy—compare England vs. back home. 6. Weight management advice in pregnancy. When you were pregnant, did you ever have any discussions about the risks of excess weight gain in pregnancy? Potential areas to discuss further: - Diet or PA-related information received during pregnancy - Source(s) of information. - Their views on the information they received. - How relevant the information was for them—advice and sources found to be most helpful/useful and reasons. - Compare England vs. back home—if pregnant in African country previously, explore any differences between information received in the UK and back home. If not pregnant in African country previously, explore their perceptions on whether the information would have been different from that received in the UK.
